# Dipeptidyl peptidase 4 interacts with porcine coronavirus PHEV spikes and mediates host range expansion

**DOI:** 10.1128/jvi.00753-24

**Published:** 2024-06-03

**Authors:** Junchao Shi, Shiyu Hu, Hanlu Wei, Le Zhang, Yungang Lan, Jiyu Guan, Kui Zhao, Feng Gao, Wenqi He, Zi Li

**Affiliations:** 1 State Key Laboratory for Diagnosis and Treatment of Severe Zoonotic Infectious Diseases, Key Laboratory for Zoonosis Research of the Ministry of Education, Institute of Zoonosis, and College of Veterinary Medicine, Jilin University, Changchun, China; 2 Key Laboratory of Organ Regeneration and Transplantation of the Ministry of Education, Jilin University, Changchun, China; 3 Department of Immunology, School of Basic Medical Sciences, Beijing Key Laboratory of Tumor Systems Biology, Institute of Systems Biomedicine, Peking University Health Science Center, Beijing, China; The Ohio State University, Columbus, Ohio, USA

**Keywords:** coronavirus, porcine hemagglutinating encephalomyelitis virus, dipeptidyl peptidase 4, spike glycoprotein, receptor recognition

## Abstract

Porcine hemagglutinating encephalomyelitis virus (PHEV), a neurotropic betacoronavirus, is prevalent in natural reservoir pigs and infects mice. This raises concerns about host jumping or spillover, but little is known about the cause of occurrence. Here, we revealed that dipeptidyl peptidase 4 (DPP4) is a candidate binding target of PHEV spikes and works as a broad barrier to overcome. Investigations of the host breadth of PHEV confirmed that cells derived from pigs and mice are permissive to virus propagation. Both porcine DPP4 and murine DPP4 have high affinity for the viral spike receptor-binding domain (RBD), independent of their catalytic activity. Loss of DPP4 expression results in limited PHEV infection. Structurally, PHEV spike protein binds to the outer surface of blades IV and V of the DPP4 β-propeller domain, and the DPP4 residues N229 and N321 (relative to human DPP4 numbering) participate in RBD binding via its linked carbohydrate entities. Removal of these *N*-glycosylations profoundly enhanced the RBD-DPP4 interaction and viral invasion, suggesting they act as shielding in PHEV infection. Furthermore, we found that glycosylation, rather than structural differences or surface charges, is more responsible for DPP4 recognition and species barrier formation. Overall, our findings shed light on virus-receptor interactions and highlight that PHEV tolerance to DPP4 orthologs is a putative determinant of its cross-species transmission or host range expansion.

**IMPORTANCE:**

PHEV is a neurotropic betacoronavirus that is circulating worldwide and has raised veterinary and economic concerns. In addition to being a reservoir species of pigs, PHEV can also infect wild-type mice, suggesting a “host jump” event. Understanding cross-species transmission is crucial for disease prevention and control but remains to be addressed. Herein, we show that the multifunctional receptor DPP4 plays a pivotal role in the host tropism of PHEV and identifies the conserved glycosylation sites in DPP4 responsible for this restriction. These findings highlight that the ability of PHEV to utilize DPP4 orthologs potentially affects its natural host expansion.

## INTRODUCTION

Coronaviruses (CoVs) are enveloped, positive-sense, single-stranded RNA viruses grouped into four genera: *Alphacoronavirus*, *Betacoronavirus*, *Gammacoronavirus*, and *Deltacoronavirus* (1, 2). Great attention has been given to betacoronaviruses because of the presence of three highly pathogenic viruses, severe acute respiratory syndrome coronavirus (SARS-CoV), SARS-CoV-2, and Middle East respiratory syndrome coronavirus (MERS-CoV), all of which have caused severe, life-threatening epidemics in recent decades (3–5). These coronaviruses are zoonotic pathogens originating from animals and cause outbreaks in the human population as the result of multiple “host jump” or spillover events. This propensity for cross-species transmission justifies close investigation of members of the genus *Betacoronavirus*.

A host jump is often related to viral species tropism, and it encounters barriers at any step in the viral life cycle (6, 7). Receptor binding can be the first step for a host jump to overcome. In most coronaviruses, the spike (S) glycoprotein on the virion envelope mediates receptor recognition following proteolytic cleavage into the S_1_ and S_2_ subunits. The S_1_ subunit is structurally organized into two distinct domains, the N-terminal domain (NTD) and the C-terminal domain (CTD), where the CTD also has a multidomain structure consisting of a receptor-binding domain (RBD) and subdomains 1 and 2. The S_2_ subunit comprises multiple α-helical segments and an antiparallel β-sheet fold at the proximal end of the viral membrane. Betacoronaviruses have evolved to adapt to broadly conserved receptors, including angiotensin-converting enzyme 2 (ACE2) (8, 9), dipeptidyl peptidase 4 (DPP4) (10), aminopeptidase N (APN) (11), carcinoembryonic antigen cell-adhesion molecule 1 (CEACAM1) (12), and some glycans (13), and even to engage homologous receptors derived from new host populations. Broad receptor engagement provides essential conditions for viruses to cross species barriers and could uniquely determine future emergence events. In the case of SARS-CoV, civets, ferrets, and raccoon dogs were identified as replicative hosts, with ≥7 amino acid substitutions observed in their respective isoforms of ACE2 compared to other infection-permissive species, suggesting that SARS-CoV seems to “tolerate” large variations in the receptor (7, 14–16). In addition to the primary receptor, multiple cell-surface molecules, known as coreceptors or attachment factors, are also required for viral entry and have been proposed to play key roles in viral propagation (17). For example, the human coronavirus OC43 (HCoV-OC43) sialoglycan-based receptor 9-*O*-acetylated sialic acid (9-O-Ac-Sia) has been suggested to be an attachment factor for HCoV-HKU1 binding to host cells (18). In addition, mutation or substitution of only a few residues on the S protein could alter receptor usage and viral adaptation, resulting in host range expansion (6, 19). Thus, characterizing sophisticated receptor recognition patterns and determining spillover events represent interesting and unresolved issues.

Porcine hemagglutinating encephalomyelitis virus (PHEV) is a neurotropic coronavirus that belongs to the genus *Betacoronavirus*, together with SARS-CoV-2, SARS-CoV, MERS-CoV, HCoV-OC43, murine hepatitis virus, and bovine coronavirus. In contrast to those of zoonotic CoVs, knowledge on the receptor-binding characteristics of animal CoVs that have not yet spilled over into human populations is lacking. With no available vaccines or therapeutics, PHEV-induced disease has raised veterinary and economic concerns (20). Clinically, PHEV can infect naïve pigs of any age and can cause subclinical to respiratory, enteric, and/or neurologic infections (21). PHEV circulating in pigs exhibits strong neurotropism in mice and rats, and there is an urgent need for surveillance of whether the virus prefers cross-species mixing and permits it in quasispecies pools. In mice, PHEV can invade the olfactory bulb intranasally, followed by rapid spread throughout the central nervous system (CNS) and survival in neural cells (22, 23). While PHEV hijacks murine neural cell adhesion molecule (NCAM) or glycans as attachment factors (18, 24, 25), their expression pattern does not entirely match the host tropism of the virus, suggesting that cell-surface cofactors are engaged in viral entry in a reservoir or intermediate species. The utilization of multiple receptors and cofactors is a predominant determinant of the host tropism and pathogenicity of viruses and is also responsible for cross-species transmission and viral fitness. However, there is limited understanding of how PHEV-host receptor interactions determine viral transmission from reservoir species to quasihosts directly or via intermediate species.

In this study, we investigated the host breadth and adaptability of PHEV and performed a challenge showing that the virus can use DPP4, also known as cluster of differentiation 26 (CD26), from pigs and mice as an attachment receptor or cofactor to enter cells. Using structure-guided mutagenesis, binding affinity studies, bioinformatics docking, and infectivity assays, we further determined that DPP4-linked N-acetyl-D-glucosamine (NAG) contributes to PHEV RBD binding. Our findings reveal the potential mechanism through which a coronavirus recognizes homologous receptors and provide insights into the cross-species transmission of PHEV. This potential utilization of DPP4 as a binding target for PHEV may offer novel insight into viral pathogenesis and help in surveillance and therapeutic strategies for overcoming the challenge of coronavirus invasion.

## RESULTS

### Host breadth of PHEV and the architecture of its spike

**Fig 1:**
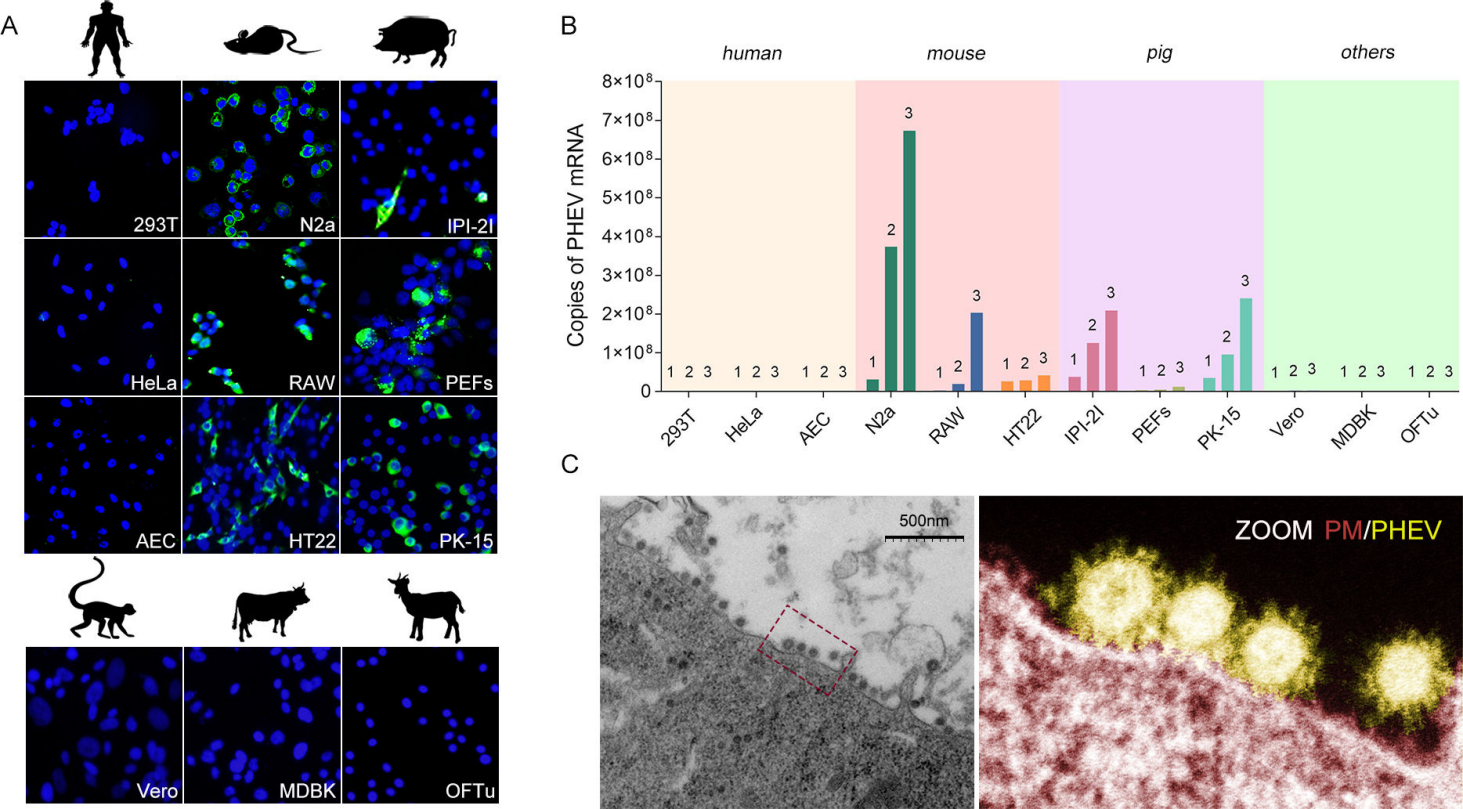
PHEV infects cell lines originating from swine and mouse species. (**A**) Immunofluorescence assay. Cells derived from different species of origin, including humans (293T, HeLa, and AEC), pigs (PK-15, IPI-2I, and PEFs), mice (N2a, HT22, and RAW), monkeys (Vero), bovines (MDBK), and ovines (OFTu), were infected with PHEV. PHEV nucleocapsid (N), green. 4',6-Diamidino-2-phenylindole (DAPI), cell nuclei. (**B**) Infection subcultures were collected at passages 1, 2, and 3 for viral load detection by quantitative reverse transcription PCR assays targeting the viral N gene. All the experiments were performed in triplicate. (**C**) Representation of virions observed in PHEV-infected N2a cells by transmission electron microscopy. The corresponding pseudocolor image is shown in the enlarged box in the right panel. Scale bar, 500 nm. PM, plasma membrane.

**Fig 2:**
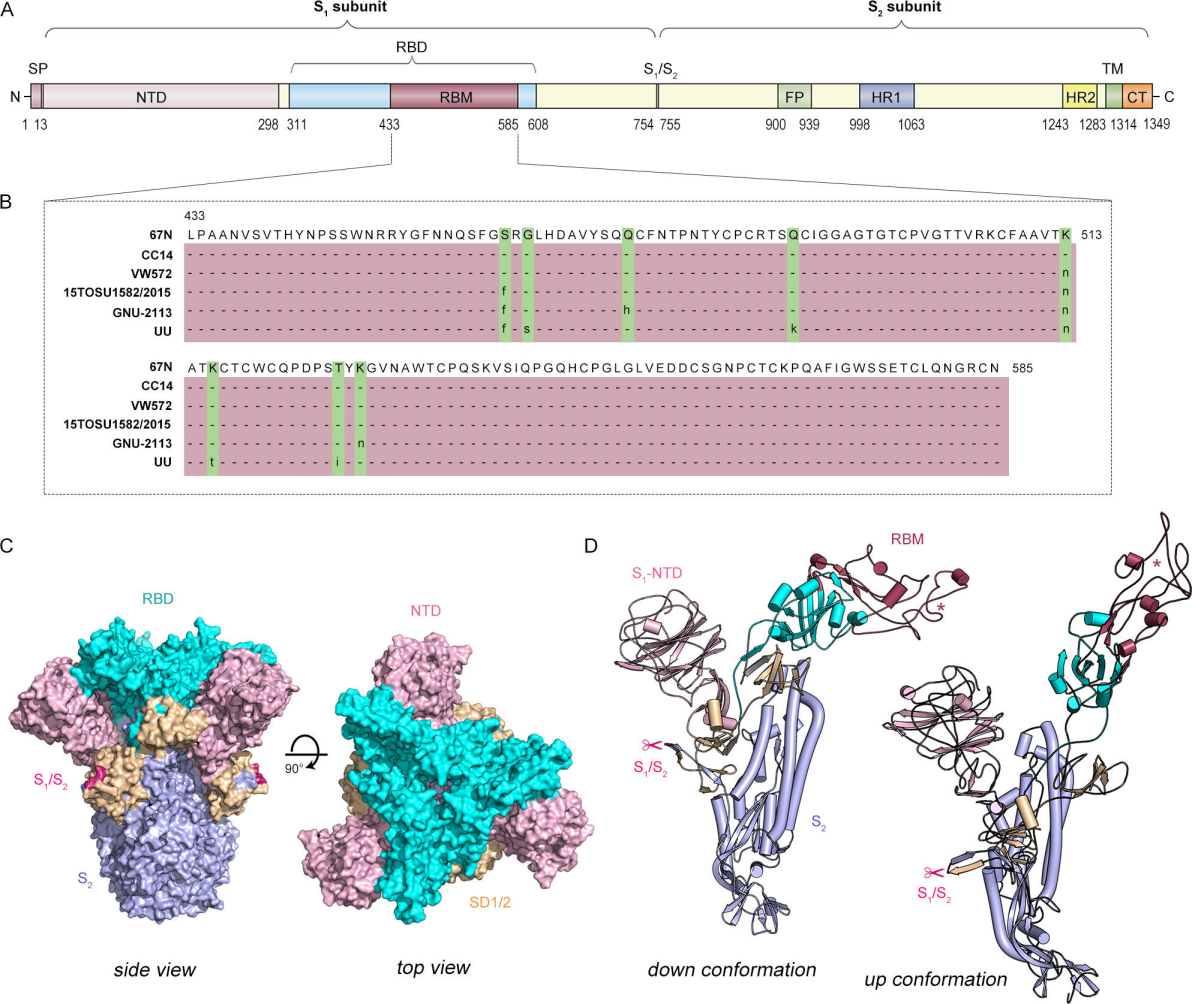
Architecture of the PHEV spike glycoprotein. (**A**) Schematic diagram of PHEV S glycoprotein organization. (**B**) Alignment of the receptor-binding motif (RBM) in six PHEV strains, namely, CC14 (GenBank: AVV64341.1), USA/15TOSU1582/2015 (GenBank: ARC95251.1), 67N (GenBank: AY078417), VW572 (GenBank: AAY68297.1), GNU-2113 (GenBank: UPA71938.1), and UU (GenBank: ASB17086.1). The mutant residues are highlighted in green. (**C**) Surface representation of the trimeric PHEV (strain CC14) S glycoprotein ectodomain in two orthogonal orientations. Three-dimensional structures are modeled by SWISS-MODEL and colored by PyMOL. The S_1_ domains NTD, RBD, and SD1/SD2 are pink, cyan, and wheat, respectively. The S_1_/S_2_ cleavage site is magenta. The S_2_ region is light blue. (**D**) Ribbon diagram depicting a view of the S monomer (upward and downward conformations) colored as in panel C. The asterisk denotes the hypervariable region. The RBM is raspberry. The scissors indicate the S_1_/S_2_ cleavage site at residues 754–755. CT, cytoplasmic tail; FP, fusion peptide; HR1/HR2, heptad-repeat; NTD, N-terminal domain; RBD, receptor-binding domain. RBM, receptor-binding motif; S1/S2, S1/S2 protease cleavage site; SD1/SD2, subdomains 1 and 2; SP, signal peptide; TM, transmembrane domain.

To visualize the infection machinery, we first assessed the host breadth and adaptability of PHEV and found that all cell lines originating from swine and mouse species were susceptible to PHEV entry (Fig. 1A). PHEV was serially passaged in triplicate on these permissive cells and showed productive infection (Fig. 1B). In light of the high neurotropism of PHEV, we used N2a cells, which are mouse neuroblasts with neuronal and amoeboid stem cell morphologies, for subsequent studies to identify putative receptors or coreceptors. Under an electron microscope, massive amounts of virions were observed attached to the surface of infected N2a cells, and viral S protruded from the viral membrane and made contact with host cells (Fig. 1C). Subsequently, the architecture of PHEV S monomers assembled into trimers was displayed. The PHEV S glycoprotein is a large type I transmembrane glycoprotein (1,348 or 1,349 amino acids) that comprises the S_1_ and S_2_ domains (Fig. 2A). Within the S_1_ subunit, a region spanning residues 433–585 constitutes the putative receptor-binding motif (RBM) according to sequence alignment of different PHEV strains from six countries: the USA, China, Canada, South Korea, Belgium, and the Netherlands (Fig. 2B). Topologically, the S_1_ subunit shows a swung-out conformation, while its RBM contains a structurally conserved core subdomain of antiparallel β-sheets and is bordered by extended loops, referred to as the hypervariable region (HVR) (Fig. 2C). In addition, models of molecular surface representation were used to predict the homotrimer structures of the PHEV S glycoprotein ectodomain (Fig. 2D).

### Dipeptidyl peptidase 4 as a candidate binding target of the PHEV spike

**Fig 3:**
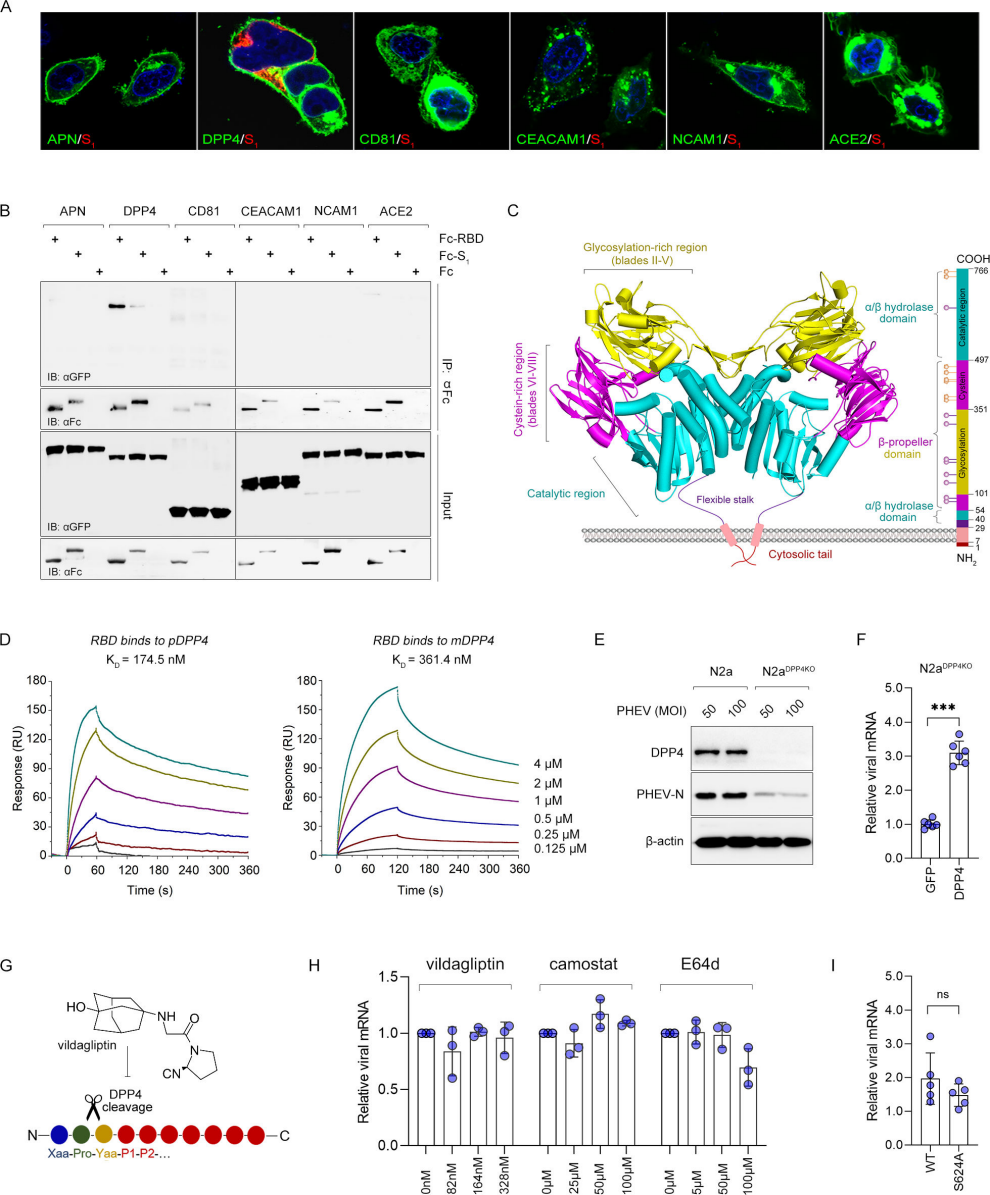
DPP4 as a candidate binding target of the PHEV spike glycoprotein. (**A**) 293T cells overexpressing the indicated protein containing a C-terminal GFP tag were inoculated with PHEV S_1_ protein for 1 h and subjected to immunostaining. (**B**) Coimmunoprecipitation assay. Recombinant Fc-tagged PHEV S_1_ or S_1_-RBD subunits were exogenously cotransfected with GFP-tagged murine APN, DPP4, CD81, CEACAM1, NCAM1, and ACE2 in 293T cells. The whole-cell lysates were immunoprecipitated and analyzed via sodium dodecyl sulfate-polyacrylamide gel electrophoresis. (**C**) Overall structure (“U”) of the homodimeric DPP4 (PDB: 5LLS) subunit, consisting of an α/β-hydrolase domain (aa 40–53 and 506–766) in cyan and an eight-bladed β-propeller domain (aa 55–496) with a glycosylation-rich region (yellow) and a cysteine-rich region (magenta). Purple sphere, *N*-glycosylation; golden pentagon, cysteine residues involved in S-bridges. (**D**) Surface plasmon resonance assay. The curves were obtained by injecting 0.125-, 0.25-, 0.5-, 1.0-, 2.0-, and 4.0-µM S_1_-RBD protein in successive cycles. (**E**) Murine DPP4 knockout N2a (N2a^DPP4KO^) cells were infected with PHEV for 48 h and evaluated via Western blotting. (**F**) GFP-tagged murine DPP4 was overexpressed in N2a^DPP4KO^ cells, and PHEV RNA replication was detected via quantitative reverse transcription PCR (qRT-PCR). Student’s *t*-test, *n* = 6. The error bars indicate SDs. ***P < 0.001. (**G**) Schematic of a DPP4 protease cleavage site. (**H**) N2a cells were incubated with vildagliptin, camostat, or E64d at the indicated concentrations for 2 h, followed by PHEV infection. Viral mRNA was measured by qRT-PCR at 48 h post infection. One-way analysis of variance, *n* = 3. The error bars indicate SDs. (**I**) PHEV mRNA in N2a cells expressing the catalytically inactive murine DPP4 mutant S624A or wild-type (WT) DPP4 was quantified at 48 h post infection. Student’s *t*-test, *n* = 5. The error bars indicate SDs. *K*_D_, dissociation constant; ns, not significant; RU, responsibility unit.

Although glycan-based receptors have been reported to support PHEV infection (18, 24), proteinaceous receptors that determine cellular entry are poorly understood and of great interest. To determine whether known receptors, such as ACE2, APN, DPP4, CEACAM1, NCAM1, and CD81, can be recognized by PHEV, an infection assay was performed and showed that only DPP4-expressing cells were susceptible to spike attachment (Fig. 3A). Furthermore, the PHEV S protein domains, i.e., S_1_ and S_1_-RBD, were strongly bound to DPP4 (Fig. 3B). DPP4 is typically anchored to the cell membrane in the form of a dimer, and its extracellular domain is further divided into a glycosylation-rich region, a cysteine-rich region, and a C-terminal catalytic region (Fig. 3C). By immobilizing soluble DPP4-IgG1 (without a transmembrane α-helix) on a biosensor surface and incubating it with monomeric S_1_-RBD as the analyte, we determined that both porcine DPP4 (pDPP4) [dissociation constant (*K*_D_) *=* 174.5 nM] and murine DPP4 (mDPP4) (*K*_D_ *=* 361.4 nM) bind to PHEV S1-RBD with moderate binding affinity (Fig. 3D). In support of this, CRISPR‒Cas9-mediated knockout of DPP4 in N2a (N2a^DPP4KO^) cells led to a sharp reduction in PHEV entry and reproduction, while overexpression of DPP4 in N2a^DPP4KO^ cells rescued viral infection (Fig. 3E and F). Although DPP4 acts as an exopeptidase by cleaving X-proline dipeptides from the N-terminus of polypeptides, we demonstrated that its activation does not affect PHEV infection by using vildagliptin (a DPP4 serine protease inhibitor), camostat mesylate (a TMPRSS2 serine protease inhibitor), or E64d (a lysosomal cathepsin-L inhibitor) (Fig. 3G and H). In addition, we mutated a key residue in the catalytic domain of DPP4, S624, which is part of the catalytic triad and vital for both exo- and endopeptidase activity. Consistent with the results of vildagliptin treatment, treatment with the catalytically inactive DPP4 mutant S624A had no significant effect on PHEV infection (Fig. 3I). Overall, we determined that DPP4 acts as a coreceptor or cofactor to facilitate PHEV infection by augmenting viral attachment and that PHEV S_1_-RBD binds to the DPP4 scaffold independently of its serine protease activation.

### Structural basis for PHEV attachment to dipeptidyl peptidase 4

**Fig 4:**
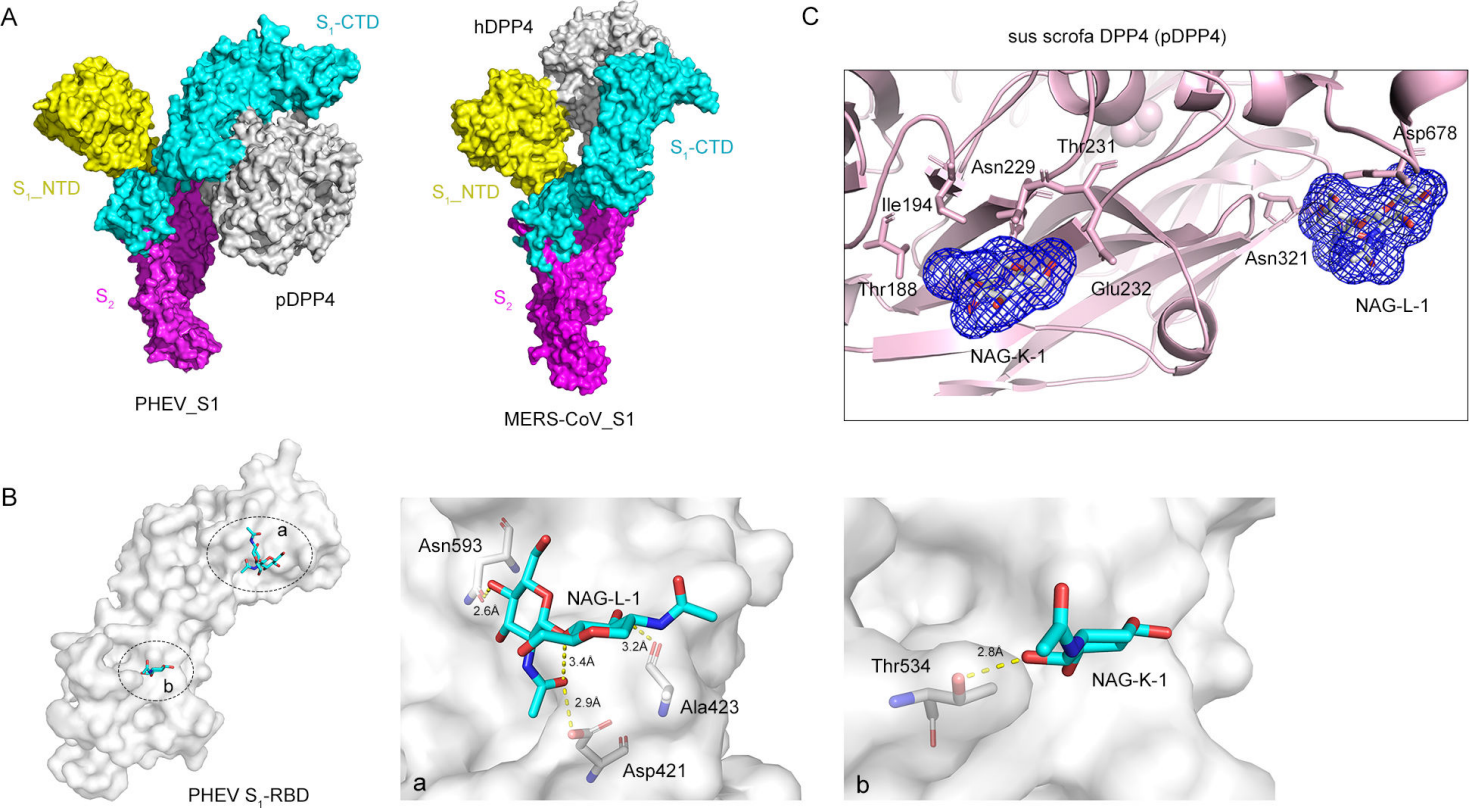
Binding interface of the PHEV S glycoprotein and DPP4. (**A**) Surface representation of PHEV S (SWISS-MODEL: 6nzk.1. A) and MERS-CoV S (PDB: 7YMZ) ectodomain monomer structures bound to porcine DPP4 (pDPP4) (PDB: 5LLS) and hDPP4 (PDB: 1TKR), respectively. The protomers are individually colored. (**B**) Surface representation of the carbohydrate moiety-binding pockets at PHEV S_1_-RBD with the surrounding key residues shown as sticks in patches a and b. The dashed yellow line indicates a hydrogen bond. (**C**) Binding interface of NAGs linked to pDPP4 N229 and N321. The pDPP4 sequence is shown as a ribbon diagram, and the residues involved are shown as pink sticks and labeled. Carbon atoms are gray; oxygen atoms are red; and nitrogen atoms are blue. NAG, N-acetyl-D-glucosamine.

**Fig 5:**
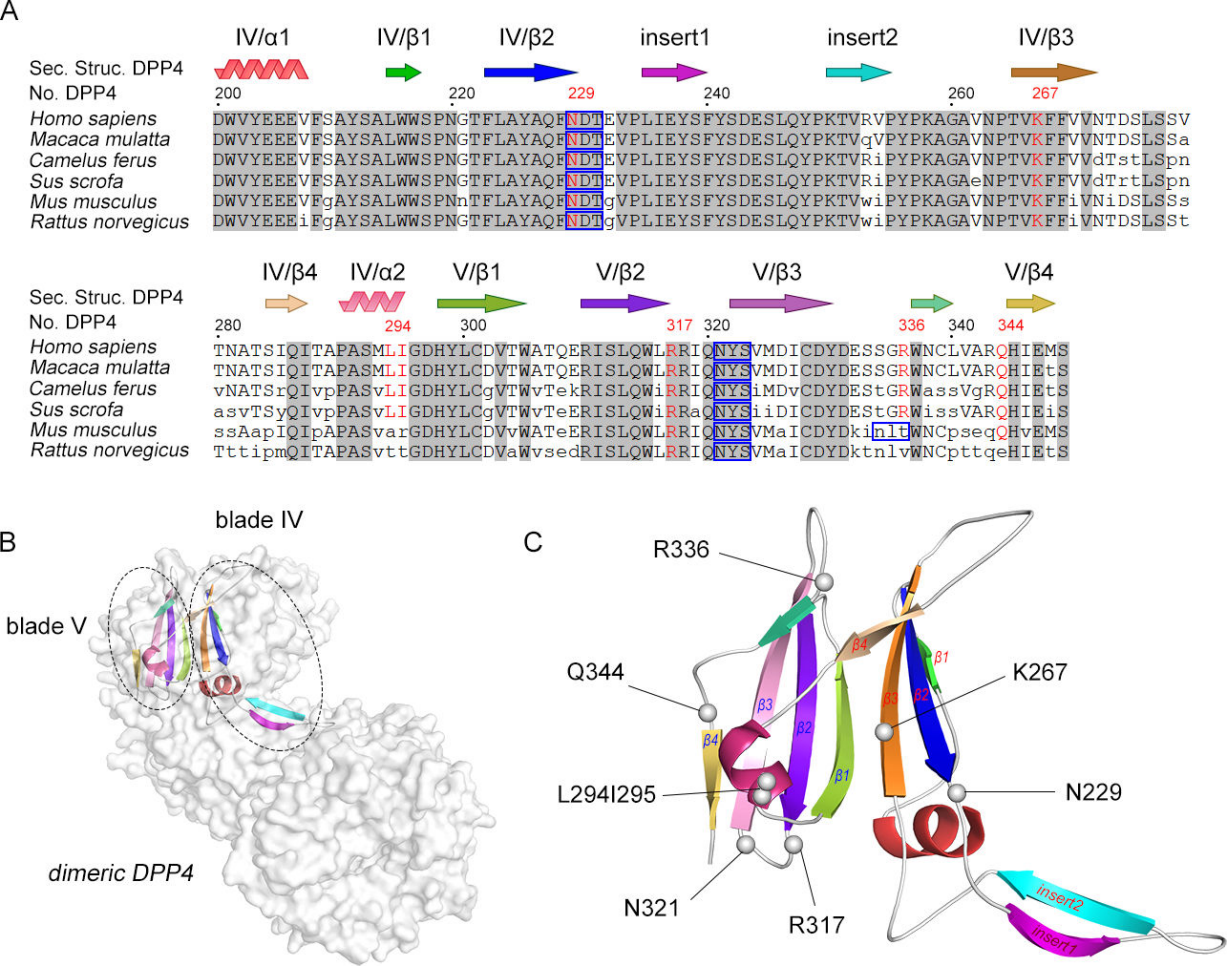
Polymorphic amino acid residues in DPP4 at the binding interface. (**A**) (Top) Topology diagram illustrating blades IV and V of the propeller structure of DPP4. β-Strands are indicated by arrows labeled with β-propeller blades in Roman numerals and β-strands in Arabic numerals. The α-helices are labeled α1 and α2. (Bottom) Alignment of the PHEV-permissive (*Sus scrofa*, *Mus musculus*, and *Rattus norvegicus*) and PHEV-resistant (*Homo sapiens*, *Macaca mulatta*, and *Camelus ferus*) DPP4 amino acid sequences. The residue numbers are relative to those of hDPP4. Amino acids engaged in the hDPP4•MERS-CoV interface are red. Nonconserved residues are in lowercase. The blue boxes represent the NXT or NXS glycosylation motif. The National Center for Biotechnology Information accession numbers are as follows: *Homo sapiens*, NP_001926.2; *Macaca mulatta*, ABC55719.1; *Camelus ferus*, XP_032335463.1; *Sus scrofa*, AY198323.1; *Mus musculus*, NM_001159543.1; and *Rattus norvegicus*, NM_012789.2. (**B**) Overall architecture of the regions of dimeric pDPP4 (PDB: 5LLS) that correspond to blades IV and V. Dimeric pDPP4 is displayed as a gray-colored molecular surface. Blades IV and V are shown as ribbons and are colored according to the topology diagram in panel A. (**C**) Zoomed-in view of blades IV and V. Location of key residues supposedly critical for coronavirus spike binding, indicated in spheres and colored gray.

To characterize the atomic interaction details, we modeled a three-dimensional reconstruction of the S glycoprotein in complex with DPP4. Interestingly, DPP4 docked to either PHEV or MERS-CoV in distinct binding domains in the S_1_-RBD (Fig. 4A and B). The predicted porcine DPP4-binding pockets (Asp421, Ala423, and Asn593; Thr534) in the PHEV S_1_-RBD are at the face distal to the binding site in the MERS-CoV S_1_-RBD, suggesting that DPP4 prefers to attach to PHEV rather than to an entry receptor. There are similarities between how DPP4 binds to the PHEV S_1_-RBD and to the MERS-CoV S_1_-RBD, both of which involve hydrogen bonds, salt bridges, and carbohydrate moieties (Table 1). Scrutinization of the binding interface revealed that PHEV S_1_-RBD interacts with a groove at the periphery of DPP4, with predicted binding sites (N229 and N321, relative to hDPP4 numbering) located in the glycosylation-rich region (blades IV and V) of the β-propeller domain of DPP4 (Fig. 4C). Alignment of the known permissive host DPP4 sequence with the nonpermissive host DPP4 sequence revealed a high degree of sequence conservation with no sequence insertion or deletion (Fig. 5A). On blades IV and V, several residues in human DPP4 (hDPP4) are engaged in intermolecular interactions at the MERS-CoV•hDPP4 binding interface (26), while two distinct porcine DPP4-linked *N*-glycosylated sites at positions 229 (NDT) and 321 (NYS) potentially contact PHEV S_1_-RBD (Fig. 5B and C). Thus, we concluded that the PHEV spike glycoprotein specifically recognizes the β-propeller domain near the ends of the arms of dimeric DPP4 for engagement.

**TABLE 1:** Details of the predicted binding interface^*a*^

Interface	Hydrogen bond			Salt bridge			Carbohydrate moiety		
	DPP4	Dist. (Å)	S_RBD	DPP4	Dist. (Å)	S_RBD	DPP4	Glycans	S_RBD
PHEV S_1_-RBD binds to pDPP4	Arg611 (NH2)	3.73	Thr637 (OG1)	Glu677 (OE1)	3.25	Arg350 (NH1)	Asn229 (ND2)* Glu232 (OE1) Thr231 (OG1)	NAG	Thr534 (OG1)
	Asn685 (ND2)	3.06	Trp642 (O)	Glu677 (OE2)	2.82	Arg350 (NH1)			
	Arg597 (NH2)	3.68	Leu645 (O)						
	Arg581 (NH2)	3.15	Tyr654 (OH)				Asn321 (ND2)* Asp678 (OD2)	NAG	Ala423 (O)
	Ile295 (O)	2.49	Lys419 (NZ)						
	Glu604 (OE2)	3.61	Gln643 (NE2)						
MERS-CoV S_1_-RBD binds to hDPP4	Leu294 (O)	3.35	Arg542 (NH1)	Lys267 (NZ)	2.84	Asp539 (OD1)	Asn299	NAG	Trp535 Glu536
	Ser334 (O)	3.61	Ser454 (OG)	Arg317 (NH1)	3.90	Asp510 (OD2)			
	Lys267 (NZ)	2.84	Asp539 (OD1)	Arg317 (NH2)	3.37	Asp510 (OD2)			
	Gln286 (NE2)	3.51	Gly538 (O)						
	Ala291 (N)	3.08	Glu513 (OE1)						
	Arg317 (NH2)	3.37	Asp510 (OD2)						
	Tyr322 (OH)	3.09	Asp510 (O)						
	Arg336 (NH2)	2.55	Tyr499 (OH)						
	Gln344 (NE2)	2.98	Glu513 (OE1)						

^*a*^ Data represent the residues that were predicted to be important for mediating the permissivity of PHEV in the pDPP4 ortholog by using PDBePIA, the protein-ligand interaction profiler, and the ZDOCK server; these residues have also been predicted and identified as important for mediating the permissivity of the hDPP4 ortholog to MERS-CoV. All residue numbers are relative to the aligning residue in hDPP4. The residues in which a glycosylation site was knocked out in the pDPP4 molecules are indicated by an asterisk (*).

### Glycosylation of dipeptidyl peptidase 4 plays a role in shielding PHEV infection

**Fig 6:**
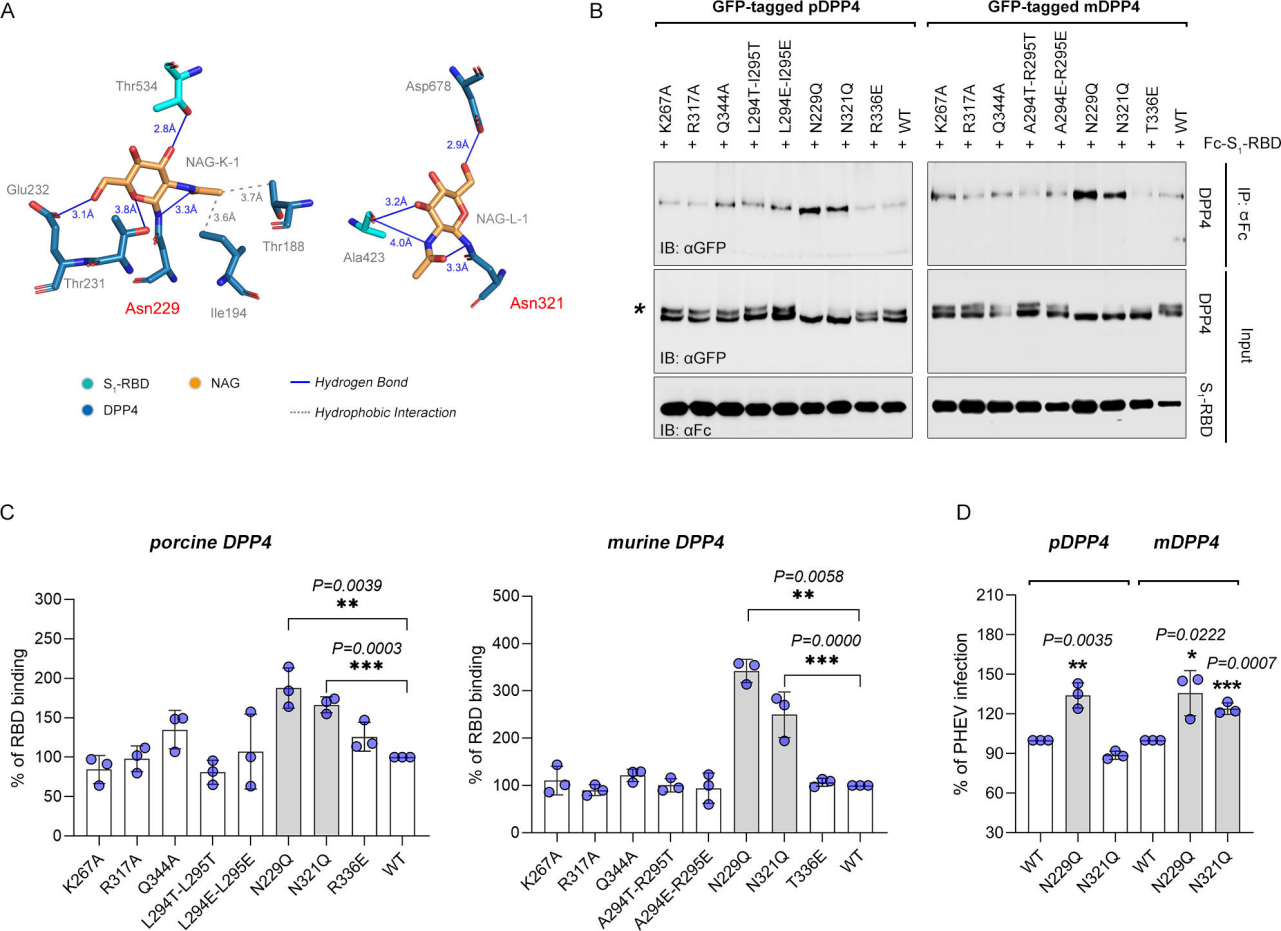
Effect of residue substitution on DPP4 binding to the PHEV S_1_-RBD. (**A**) Glycoside residues bound to Asn229 and Asn321 of pDPP4 interact with Thr534 and Ala423 of the PHEV S_1_-RBD, respectively. The carbon atoms of DPP4 are indigo; the carbon atoms of S_1_-RBD are cyan; and the carbon atoms of NAG are orange. Oxygen is red, and nitrogen is blue. (**B**) Coimmunoprecipitation analysis of copurified complexes of wild-type (WT) or mutant forms of GFP-tagged pDPP4 or murine DPP4 (mDPP4) and soluble Fc-tagged PHEV S_1_-RBD in 293T cells. The larger bands marked with an asterisk (*) represent glycosylated DPP4. (**C**) Quantitative analysis of the binding of the RBD to pDPP4 or mDPP4. Student’s *t*-test, *n* = 3. The error bars represent the SDs. **P < 0.01, ***P < 0.001. (**D**) qRT-PCR analysis of PHEV mRNA in 293T cells expressing WT or mutant DPP4. Student’s *t*-test, *n* = 6. The error bars represent the SDs. ***P < 0.001.

Considering the crucial residues at the binding interface of the MERS-CoV•hDPP4 complex (27), we generated a panel of DPP4 mutants, as indicated in Fig. 5C. We first assessed the functional relevance of the carbohydrate moiety. As shown in Fig. 6A, N229-linked NAG-K-1 contacts T534 of PHEV S_1_-RBD and forms intermolecular hydrogen bonds and hydrophobic interactions, whereas two hydrogen bonds are formed between N321-linked NAG-L-1 and A423 of PHEV S_1_-RBD. The N229Q and N321Q substitutions in which an NXT (or NXS) glycosylation motif was knocked out resulted in a significantly enhanced interaction with PHEV S_1_-RBD (Fig. 6B and C). Removal of glycosylation in DPP4 appears to increase virus invasion, but this effect was not seen in other mutant groups (Fig. 6D). These results suggest that *N*-glycosylation not only mediates contact between DPP4-linked carbohydrate entities and viral spike but also acts as a shield against PHEV infection.

Given that two residues, L294 and I295, near the MERS-CoV S_1_-RBD amino acid pair and form a hydrophobic center at the interface (27), we also evaluated the L294 mutant in the context of the I295 residue of pDPP4. With respect to the L294EI295E and L294TI295T mutations, the elimination of unfavorable free charges at these residues had no significant effect on receptor-binding capacity (Fig. 6B and C). Notably, the murine DPP4 receptor contains two substitutions (Leu-to-Ala and Ile-to-Arg) at positions 294 and 295 (relative to hDPP4 numbering) and is therefore devoid of the key hydrophilic interactions rendered by the leucine residue. In addition, disruption of polar contacts (hydrogen bonds or salt bridges) at the K267, R317, and Q344 sites had no effect on PHEV S_1_-RBD binding (Fig. 6B and C), although these interactions have been identified as the primary factor underlying the permissiveness of hDPP4 during MERS-CoV invasion (27). These findings suggest that DPP4-linked glycosylations at positions 229 and 321 are responsible for regulating its ability to bind to viral RBD and play a role in shielding PHEV infection.

### Interspecies adaptation of PHEV to dipeptidyl peptidase 4 orthologs

**Fig 7:**
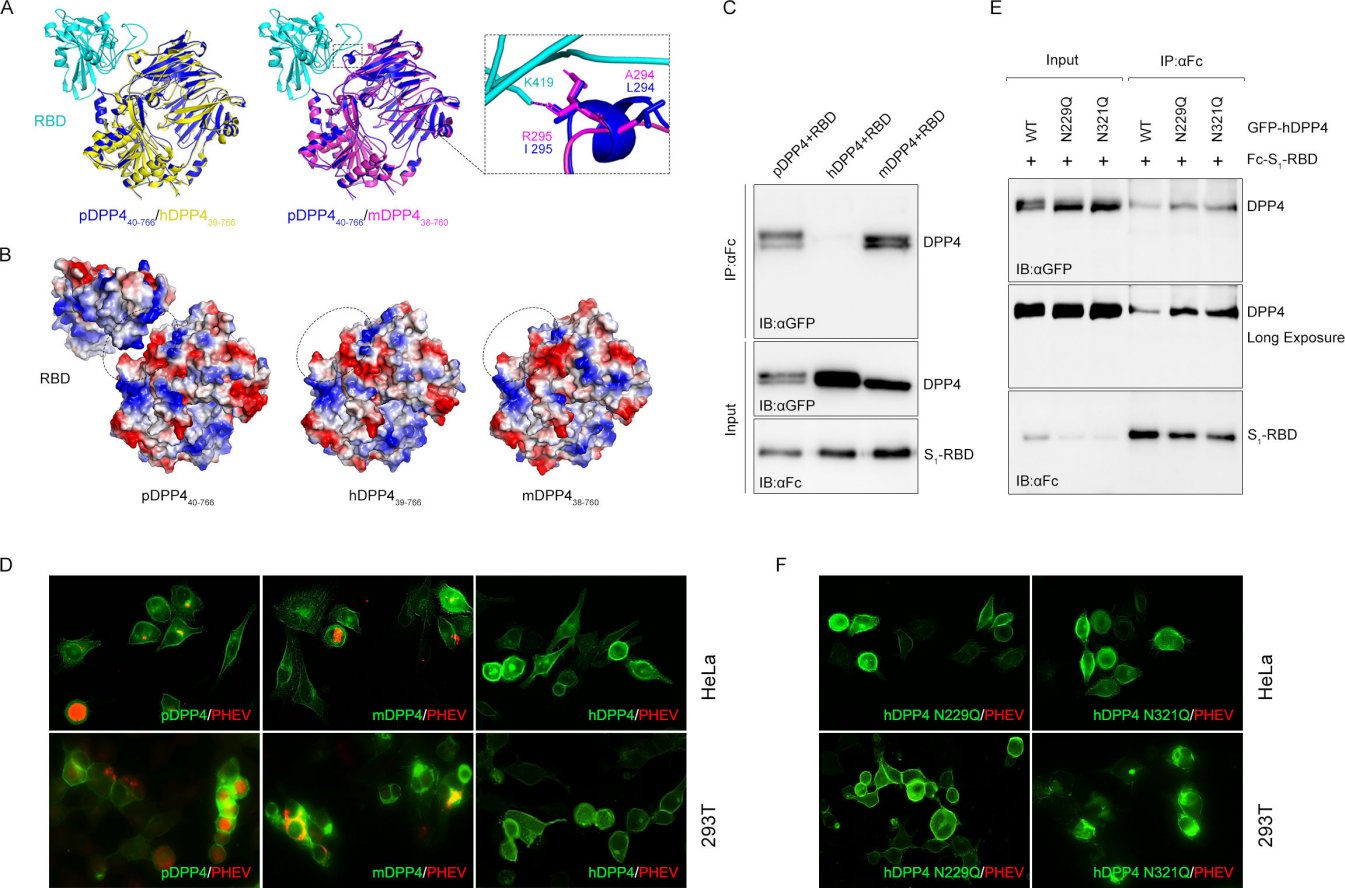
Comparison among DPP4 orthologs interfacing with the PHEV S_1_-RBD. (**A**) Structural comparison of threaded molecules of hDPP4 (yellow; PDB: 4 L72, amino acids 39–766) and mDPP4 (purple; AlphaFold: P28843, amino acids 38–760) to pDPP4 (blue; PDB: 5LLS, amino acids 40–766), overlaid on complex with the PHEV S_1_-RBD (cyan, amino acids 334–602). (**B**) Surface charges of DPP4 orthologs and PHEV S_1_-RBD. Blue indicates a positive charge, and red indicates a negative charge. (**C**) Coimmunoprecipitation (Co-IP) analysis of copurified complexes of GFP-tagged hDPP4, pDPP4, or mDPP4 and Fc-tagged PHEV S_1_-RBD in 293T cells. (**D**) Immunostaining assay. HeLa or 293T cells expressing GFP-tagged DPP4 orthologs were infected with PHEV, fixed, and probed with an anti-PHEV N polyclonal antibody. (**E**) Co-IP analysis of hDPP4 mutants and PHEV S_1_-RBD in 293T cells. (**F**) Immunostaining analysis of HeLa or 293T cells overexpressing GFP-hDPP4 mutants and infected with PHEV.

To better understand the interspecies transmission of PHEV, we compared the overall structures (available crystal structure or predicted model) of DPP4 orthologs that represent a broad host breadth, including pDPP4, mDPP4, and hDPP4. Apart from two mutations at positions 294 and 295 within the last α-helix region of blade IV of mDPP4, these homologous structures are predicted to have a backbone topology highly similar to that of pDPP4 at a large surface area (Fig. 7A). Consistent with the binding assay results shown in Fig. 6B, we suggest that the hydrophobic core, which is essential for the binding interface of the MERS-CoV•hDPP4 complex, is not required for PHEV S_1_-RBD binding. Electrostatic potential analysis revealed the negative charges of permissive pDPP4 and mDPP4 at the binding interface and that a small hydrophobic depression in the DPP4s further cradles the bulged loop in the RBD HVR (Fig. 7B). The PHEV S_1_-RBD is positively charged, making it complementary to pDPP4. A similar scenario was simultaneously observed for hDPP4, which is resistant to PHEV (Fig. 7B), implying that DPP4 orthologs can likely act as backbones supporting PHEV fitness by increasing binding affinity. Furthermore, transient transfection of porcine and murine DPP4-expressing cells promoted infection after the cells were able to interact with PHEV S_1_-RBD (Fig. 7C and D). Unexpectedly, human DPP4 was also immunoprecipitated by the RBD, although with much lower efficiency than that of the susceptible host receptor (Fig. 7C). Removal of the N229- and N321-linked glycosylations in hDPP4 enhanced the binding of RBD to hDPP4 (Fig. 7E), but they did not support PHEV infection in human cells (Fig. 7F). It is plausible that glycosylation of DPP4 is necessary but not sufficient for PHEV infection. Taken together, we concluded that glycosylation in the host receptor DPP4, rather than structural differences or interface surface charges, is involved in forming a species barrier for PHEV infection.

## DISCUSSION

Coronavirus pandemics represent a significant threat to global public health and are strongly fueled by species jumping, a mechanism for coronavirus persistence and survival (28). While numerous swine coronaviruses, such as porcine epidemic diarrhea virus, transmissible gastroenteritis virus, porcine deltacoronavirus, and emerging swine acute diarrhea syndrome coronavirus, have been surveilled in the past decade, PHEV continues to be less researched but is a good prototype pathogen for investigating the neuropathological pathogenesis of acute coronavirus infection (29, 30). A clinical PHEV outbreak was first described in 1957 in Canada and subsequently worldwide (31–36). PHEV is transmitted primarily through respiratory droplets and close contact, where it enters the CNS via the peripheral nervous or the olfactory system (22, 37, 38). The utilization of multiple receptors and cofactors is a predominant determinant of the host tropism and pathogenicity of viruses and is also responsible for cross-species transmission and viral fitness. However, there is limited understanding of how virus-host receptor interactions determine viral transmission from reservoir species to quasihosts directly or via intermediate species.

In this paper, we identified DPP4/CD26 as an attachment receptor or cofactor for PHEV infection through functional approaches, binding affinity studies, bioinformatics docking, and structure-guided mutagenesis. DPP4 (EC3.4.14.5) is a type II integral transmembrane glycoprotein that is dimeric, with each monomeric ectodomain structurally comprising two domains, an α/β-hydrolase domain and an eight-bladed β-propeller. As a multifunctional serine exopeptidase, DPP4 is highly conserved among mammals and plays a major role in glucose metabolism, immunological processes, and inflammatory diseases (27, 39). However, we focused more on DPP4 as a receptor that binds to the external S glycoproteins of MERS-CoV and Ty-BatCoV, initiating coronavirus entry into host cells (10, 40). Animal studies have shown that rhesus macaques but not hamsters, ferrets, or mice are susceptible to MERS-CoV infection. The presence of a species barrier should therefore be attributed to the inability of MERS-CoV to recognize DPP4 from these species, which harbor too many mutations in the RBD-binding region (7, 41). The affinity of the MERS-CoV S RBD for human DPP4 is ~17 nM, but low-affinity goat and bat receptors can also hypersensitize cells to infection when an S-cleaving protease is present (26, 42). This notion is supported by PHEV preferentially infecting cells expressing DPP4 orthologs, which have a ~3-fold greater affinity for pDPP4 than for mouse DPP4 (mDPP4). While we compared the overall structures of orthologous DPP4 strains that were classified as PHEV permissive (pig, mouse, or rat) or nonpermissive (human, camel, or macaque), each structure was predicted to have a backbone topology highly similar to that of pDPP4. These observations raise the possibility that binding affinity to the DPP4 receptor may not be the most critical factor for adaptation to new species and that receptor variation may act as a backbone supporting host jumps or spillover events by increasing the binding affinity.

There are two major domains in the S_1_ subunit of the coronavirus S glycoprotein: the N-terminal domain (S_1_-NTD) and the C-terminal domain (S_1_-CTD). Both of these proteins potentially bind host receptors and function as receptor-binding domains (RBDs). S_1_-NTD is responsible for binding glycan chains on glycoproteins and lipids (43–45). Our previous work and a recent publication demonstrated that PHEV S_1_ domain A recognizes cell-surface glycans, such as sialic acid, heparan sulfate, and 9-O-Ac-Sia, to support infection (18, 24). In contrast to S_1_-NTD, S_1_-CTD appears to bind exclusively to proteinaceous receptors. Here, we determined that PHEV S_1_-CTD recognizes the β-propeller region near the ends of the arms of the U-shaped DPP4 dimer away from the peptidase catalytic site. In contrast to the strong polar contact (H-bond/salt-bridge or multiple van der Waals contacts) network-dominated interactions of the MERS-CoV•hDPP4 complex (26), the PHEV RBD has a gently convex surface and binds to DPP4 via a carbohydrate moiety. The interaction is predominantly mediated by residue side chains, including RBD T534 with DPP4 N229-linked NAG and RBD A423 with DPP4 N321-linked NAG (relative to hDPP4 numbering). Glycosylation at the N229 and N321 sites may act as a shield against the direct binding of DPP4 to the RBD at the binding interface. After removing the NXT glycosylation motif by introducing the N229Q substitution into mDPP4, we observed an increase in both the RBD-mDPP4 interaction and PHEV infection. For MERS-CoV, removal of the NLT mDPP4 putative glycosylation site (N328-L329-T330) is responsible for regulating the ability of mDPP4 to function as a functional receptor (46, 47), further suggesting that glycosylation can act as a determinant of DPP4-mediated host range expansion. A similar scenario was observed for the APN orthologs. Porcine and feline APN orthologs are glycosylated, suggesting that porcine and feline coronaviruses utilize them as functional receptors, whereas the glycosylation of human APN was found to confer resistance to HCoV-229E infection (48). In other words, glycosylation is a determinant of receptor species specificity for numerous coronaviruses.

The atomic details at the binding interface provide structural insight into virus and receptor interactions, which could guide the development of therapeutics and vaccines against PHEV infection. Alignment of amino acid residues within blades IV and V revealed a minimal subset of three amino acid changes between human and mouse DPP4; i.e., A294, R295, and T336 in mDPP4 corresponded to L294, I295, and R336 in hDPP4, respectively. In the MERS-CoV•hDPP4 binding interface, the bulged helix in DPP4 properly positions the hydrophobic residues L294 and I295 near the RBD amino acids, forming a hydrophobic core (26, 27). This interaction, however, is altered in PHEV, potentially making the hydrophobic center less amenable to interacting with the PHEV RBD. As shown by the results for the mutated mDPP4 proteins A294T-R295T and A294E-R295E, these two hydrophobic residues were not sufficient to affect PHEV RBD binding activity or viral entry efficiency. Considering both the RBD structure and the binding mode with receptors, PHEV differs from MERS-CoV. Nevertheless, we noted that in the RBD-receptor complex structures of both PHEV and MERS-CoV, the binding interfaces involve the glycosylation state of DPP4. Investigating the contribution of the carbohydrate moiety to the virus-receptor interaction for PHEV would therefore be interesting in the future.

In summary, our study revealed that the MERS-CoV receptor DDP4 acts as a candidate binding target or coreceptor for PHEV, highlighting how viral spikes recognize receptor-N-linked carbohydrate entities at the atomic level to increase binding affinity. Hence, PHEV has adapted to exploit weak multivalent interactions and display considerable redundancy and flexibility as potentiating evolutionary advantages in cross-species transmission. This model provides new insight into the interaction between PHEV and the host; however, the crystal structure underlying the native receptor-bound PHEV spike RBD has yet to be determined. Future studies in this area can provide new knowledge about the host tropism, spread, and pathogenicity of PHEV and help develop surveillance and therapeutic strategies for coronavirus outbreaks.

## MATERIALS AND METHODS

### Virus, cell lines, and antibodies

The PHEV strain used in the study was HEV 67N (GenBank accession number AVV64341.1). The virus was produced in N2a cells and stored at −80°C until use. HeLa, 293T, AEC, RAW 264.7, HT22, PK-15, IPI-2I, PEFs, Vero, MDBK, and OFTu cells were stored in the laboratory. All the cell lines were cultured in Dulbecco’s modified Eagle medium (DMEM; Gibco, USA) supplemented with 10% fetal bovine serum (FBS), 1% penicillin/streptomycin, and 2% L-glutamine at 37°C with 5% CO_2_. The anti-PHEV-nucleocapsid polyclonal antibody was stored in the laboratory. Horseradish peroxidase (HRP)-conjugated goat anti-rabbit IgG, HRP-conjugated goat anti-mouse IgG, GAPDH monoclonal antibody, β-actin monoclonal antibody, GFP monoclonal antibody, anti-mouse Alexa Fluor 488-conjugated IgG, and anti-rabbit Alexa Fluor 594-conjugated IgG were purchased from CST. The CD26 antibody C-terminus was purchased from Affinity. Goat anti-human IgG Fc (HRP) and goat anti-human IgG Fc (DyLight 650) were obtained from Abcam.

### Plasmids

The full-length gene coding region sequences of ACE2 (GenBank: NM_001130513.1), DPP4 (GenBank: NM_010074.3), APN (GenBank: NM_008486.3), CEACAM1 (GenBank: NM_011926.2), CD81 (GenBank: NM_133655.2), and NCAM1 (GenBank: NM_010875.4) were subcloned and inserted into the pcDNA3.1 vector with a C-terminal GFP tag. DPP4 mutants were generated by overlap PCR mutagenesis. PHEV S_1_-RBD and PHEV S_1_ were subcloned and inserted into the pFUSE-hIgG1-Fc vector with a C-terminal Fc tag. A CRISPR/Cas9-mediated DPP4 knockout plasmid (PX459-DPP4) was constructed by subcloning the gRNA-1 sequence (sense: 5ʹ- CACCGGCACTCCTGTGTCGTTAAAC-3ʹ, antisense: 5ʹ- AAACGTTTAACGACACAGGAGTGCC-3ʹ) and the gRNA-2 sequence (sense: 5ʹ- CACCGAGAGTAGGACTTGACCCGAA-3ʹ, antisense: 5ʹ- AAACTTCGGGTCAAGTCCTACTCTC-3ʹ) into the pSpCas9(BB)-2A-Puro (PX459) vector.

### Virus infection

The PHEV strain used in the study was CC14 (GenBank: AVV64341.1), and it was stored in the He laboratory. The virus was produced in N2a cells and stored at −80°C until use. The cells were preincubated with PHEV at 100 TCID_50_ at 37°C for 1 h, washed with PBS, and maintained in DMEM supplemented with 2% FBS before further incubation for 24–72 h.

### Plasmid transfection

The cells were cultured in six-well plates. Three microliters of Lipofectamine 3000 reagent was diluted in 100 µL of Opti-MEM; 2 µL of plasmid containing a gene with a GFP or Fc tag (1 µg/µL) and 4 µL of P3000 reagent were mixed with 100 µL of Opti-MEM. Following incubation for 10 min, the DNA-lipid complex was added to the cells, which were subsequently incubated at 37°C with 5% CO_2_. Lysates of the transiently transfected cells were collected and analyzed via Western blotting (WB) at 72 h post transfection. An empty plasmid was used as a transfection control.

### Quantitative RT-PCR

Total RNA was extracted from treated cells using TRIzol (Takara, Japan) according to the manufacturer’s instructions, and reverse transcription was performed with an RT-PCR Kit (TransGen, China). The expression of PHEV genes was determined by two-step quantitative reverse transcription PCR (qRT-PCR) and analyzed using the 2^−ΔΔCT^ method. The sequences of the primers used for qRT-PCR were as follows: PHEV sense, 5ʹ-AGCGATGAGGCTATTCCGACTA-3ʹ; PHEV antisense, 5ʹ-TTGCCAGAATTGGCTCTACTACG-3ʹ; GAPDH sense, 5ʹ-CTCAACTACATGGTCTACATGTTC-3ʹ; and GAPDH antisense, 5ʹ- ATTTGATGTTAGTGGGGTCTCGCTC-3ʹ.

### WB

The cells were collected and lysed in radioimmunoprecipitation assay (RIPA) buffer on ice for 30 min. Subsequently, 5× loading buffer was added to the cell lysates, and the mixture was denatured at 100°C for 10 min. After separation by 8% sodium dodecyl sulfate-polyacrylamide gel electrophoresis, the proteins were transferred to polyvinylidene difluoride membranes. After blocking with 5% nonfat milk at 37°C for 1 h, the membranes were washed with phosphate buffered saline with Tween-20 (PBST) and incubated with the indicated primary antibodies (1:1,000) at 4°C overnight. After washing three times, the membranes were incubated with HRP-conjugated secondary antibodies (1:10,000) at 37°C for 1 h. The signals were visualized with an enhanced ECL system.

### CRISPR/Cas9-mediated DPP4 knockout

A CRISPR/Cas9-mediated DPP4 knockout plasmid (PX459-gRNA DPP4) was constructed by subcloning the gRNA sequence (sense: 5ʹ- CACCGGCACTCCTGTGTCGTTAAAC-3ʹ; antisense: 5ʹ- AAACGTTTAACGACACAGGAGTGCC-3ʹ) into the pSpCas9(BB)−2A-Puro (PX459) vector. N2a cells were cultured in six-well plates, transfected with the PX459-gRNA DPP4 plasmid using Lipofectamine 3000 transfection reagent, and further screened with puromycin dihydrochloride (Beyotime, China) at 5 µg/mL in DMEM containing 6% FBS. After 72 h of transfection, DPP4 expression was determined using RT-qPCR and WB.

### Indirect immunofluorescence assay

The cells were seeded in a 12-well plate with coverslips, washed with PBS, fixed with 4% paraformaldehyde at room temperature for 10 min, and then permeabilized with 0.5% Tween-20. After washing three times with PBST, the coverslips were blocked with 5% bovine serum albumin (BSA) at 37°C for 1 h, and 50 µL of primary antibody (1:100) was added at 4°C overnight. Next, the coverslips were washed three times and incubated with 50 µL of secondary antibody (1:500) at 37°C for 30 min. Thereafter, the cells were washed three times and stained with 1-µg/mL DAPI for 10 min at room temperature. The images were collected using a fluorescence microscope or confocal microscope FV1200MPE (Olympus, Japan).

### Coimmunoprecipitation

A coimmunoprecipitation assay was performed to evaluate the interaction between the receptor protein and the PHEV spike protein. Briefly, 293T cells were grown in six-well plates and cotransfected with expression plasmids encoding PHEV S domains and receptors. Forty-eight hours after transfection, the cells were washed with PBS, lysed in 200 µL of RIPA buffer containing phenylmethanesulfonyl fluoride (PMSF) on ice for 20 min, and subsequently centrifuged at 12,000 × *g* for 10 min. Subsequently, 5 µL of Pierce protein A/G agarose (Thermo Fisher, USA) was mixed with 150 µL of cell lysates and incubated at 4°C overnight, while the remaining 50 µL of the cell lysates was added to 5× loading buffer and boiled for 10 min to analyze comparable total protein levels. Then, the beads were centrifuged at 1,500 × *g* for 3 min and washed three times with PBS. Finally, the supernatants were removed, and loading buffer was added. After boiling for 5–10 min, the eluted proteins were analyzed via Western blotting as described above. To account for variations in transfection efficiency and sample processing, the signal intensities of the protein bands were quantified with β-actin or Fc. The images were analyzed using ImageJ.

### Structural modeling

Modeling in SWISS-MODEL, the PHEV spike protein in the RBD downconformation was constructed using the human coronavirus spike surface glycoprotein structure (SMTL ID: 6nzk.1. A) as a template, while the PHEV spike protein in the RBD upconformation was constructed from the human coronavirus HKU1 spike glycoprotein structure (SMTL ID: 8opo.1) as a template. The murine and macaque DPP4 models were predicted and generated with the AlphaFold Protein Structure Database. For each species, the structure of the RBD-DPP4 complex was generated by merging the RBD with DPP4, which was subsequently subjected to electrostatic potential analysis using the PDB2PQR server and the adaptive Poisson-Blotzmann Solver tool extension in PyMol. The binding pocket, energy, and interface of the RBD-DPP4 complex were predicted by using PDBePIA, the protein-ligand interaction profiler, and the ZDOCK server. All structural figures were made with PyMol.

### Surface plasmon resonance

Surface plasmon resonance experiments were performed using a BIAcore X-100 system at room temperature with CM5 sensor chips (GE Healthcare). Porcine or murine DPP4 proteins were immobilized on the chips via amine coupling. PHEV spike RBD proteins (i.e., 4.0, 2.0, 1.0, 0.5, 0.25, and 0.125 µM) were titrated in HEPES buffer (0.01-M HEPES, 0.15-M NaCl, 0.5% vol/vol surfactant P20, 3-mM EDTA, pH 7.4) and subsequently injected. After each cycle, the sensor surface was regenerated using 10-nM glycine-HCl (pH 3.0). Measurements from the reference flow cell (immobilized with BSA) were subtracted from the experimental values. The kinetic data were analyzed with Biacore X100 Evaluation software using the steady-state affinity model.

### Statistical analysis

All the data are presented as the means ± SDs or ±SEMs. All the statistical analyses were performed with GraphPad Prism v.8.2.1. *P* values were obtained from unpaired Student’s *t*-tests or one-way analysis of variance. Statistical significance is indicated by * (*P* < 0.05), ** (*P* < 0.01), *** (*P* < 0.001), and **** (*P* < 0.0001). All the experiments were repeated at least three times.

## Data Availability

The original data obtained in the study are included in the article. Further inquiries can be directed to the corresponding author.
